# Case report: Rare floating gallbladder torsion in a child

**DOI:** 10.3389/fmed.2024.1407716

**Published:** 2024-05-30

**Authors:** Haiyang Ren, Hao Liu, Xiurui Liu, Hua Wei, Ping Tian

**Affiliations:** ^1^Department of General Surgery, Linyi Maternity and Child Health Care Hospital, Linyi, Shandong, China; ^2^Department of Gastroenterology, Linyi People’s Hospital, Linyi, Shandong, China

**Keywords:** gallbladder torsion, child, acute abdomen, floating gallbladder, laparoscopic cholecystectomy

## Abstract

Gallbladder Torsion (GT) refers to serious biliary emergencies caused by the torsion of the gallbladder on its mesentery along the axis of the cystic duct and cystic artery. It is very rare, especially in children. The clinical data of a child with floating gallbladder torsion who was treated in our hospital on March 14, 2024, were analyzed. A 6-year-old girl presented with abdominal pain and vomiting. Physical examination showed a mass in the right middle abdomen. Laboratory tests showed normal liver biochemical function and white blood cells. The benign lesion was considered by color Doppler ultrasound and CT, and the floating torsion of the gallbladder was diagnosed by MRCP and laparoscopic exploration. The child was treated with laparoscopic cholecystectomy (LC) and recovered well after the operation.

## 1 Introduction

GT is rare and occurs due to torsion of the gallbladder on its mesentery along the axis of the cystic duct and cystic artery (1). Acute GT is very rare, with a rough statistical incidence of 1/365520 (2). So far, less than 600 cases have been reported worldwide (3), most of them are elderly and female adults. The incidence of females is higher than that of males, about 5:1 (3). It is more rare in children. There are less than 60 cases of pediatric GT reported in Chinese and foreign literature. The incidence of boys and girls is about 2.5:1 (4), and most of them are free GT. Due to the lack of specificity in its clinical manifestations and diagnosis, it is often missed or misdiagnosed, and the misdiagnosis rate is more than 90% (5). On March 14, 2024, a 6-year-old girl with free GT was admitted to our department. Now, the diagnosis and treatment process were retrospectively analyzed and the literature was reviewed for learning and discussion.

## 2 Case description

### 2.1 Patient information

The child, 6 years old, presented with “abdominal pain with vomiting for 1 day.” One day ago, the child had abdominal pain without obvious inducement, mainly for periumbilical pain, which continued, and paroxysmal aggravation, accompanied by vomiting, mostly with abdominal pain. After vomiting, the abdominal pain was slightly relieved, and the vomiting was gastric content, non-jet, so we came to our hospital for a clear diagnosis and treatment.

### 2.2 Clinical findings

Physical examination: the whole abdominal abdomen wall is soft, and the right middle abdomen is fixed with tenderness, palpable mass, about 4.0 cm × 3.0 cm in size, oval, tough, smooth, with poor mobility. The gallbladder was not palpable, and the Murphy sign was uncooperative.

### 2.3 Timeline

The child had no similar medical history.

### 2.4 Diagnostic assessment

**Figure 1:**
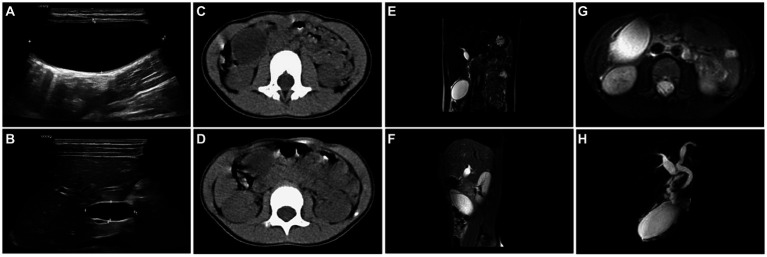
**(A)** Abdominal ultrasound showed a cystic area of 7.2 cm × 3.1 cm × 3.8 cm in the right upper abdomen, with clear boundaries and sound transmission. **(B)** Abdominal ultrasound showed a cystic area of 2.7 cm × 1.1 cm × 1.4 cm at the gallbladder fossa, with clear boundaries and sound transmission. **(C,D)** Abdominal CT showed an oval low-density shadow, about 65 mm × 26 mm × 30 mm in size, with a clear boundary in the right upper abdomen (lower edge of the liver). The gallbladder was not clear, and cystic shadow was seen in the gallbladder fossa area, and the common bile duct was slightly dilated. **(E)** Coronal T2-W MRI showed that there was no normal gallbladder and cystic duct in the gallbladder fossa area, an ovoid cystic high signal was seen in the lower layer of the gallbladder fossa, and a tortuous tubular structure was seen in the anterior superior layer to the direction of the gallbladder fossa. **(F)** Sagittal T2-W MRI showed that there was no normal gallbladder and cystic duct in the gallbladder fossa area, and the oval cystic high signal was observed at the level below the gallbladder fossa. **(G)** Horizontal T2-W MRI showed an ovoid cystic high signal (52 mm × 30 mm) below the gallbladder fossa, and the fluid signal was seen around the cystic structure. **(H)** MRCP showed a tortuous tubular structure above the gallbladder, which was highly suspected of gallbladder torsion.

Emergency B-ultrasound examination revealed a cystic area of 7.2 cm × 3.1 cm × 3.8 cm in the right upper abdomen, with clear boundaries and sound transmission (Figure 1A). A cystic area of 2.7 cm × 1.1 cm × 1.4 cm could be detected at the gallbladder fossa, with clear boundaries and sound transmission inside (Figure 1B). Further emergency CT examination showed an oval low-density shadow of about 65 mm × 26 mm × 30 mm in the right upper quadrant of the abdomen (lower edge of the liver), with a clear boundary and an internal CT value of about 8 HU. The gallbladder was not clear, the cystic shadow was seen in the gallbladder fossa area, and the common bile duct was slightly dilated, which was considered to be a benign lesion (Figures 1C,D). On the first day of admission, blood routine + C-reactive protein: white blood cell 8.26 × 10^9^/L, C-reactive protein 0.5 mg/L. Emergency biochemical tests showed alanine aminotransferase 13.9 U/L, aspartate aminotransferase 31.0 U/L, aspartic/alanine aminotransferase ratio 2.23, total bilirubin 7.7 μmol/L, direct bilirubin 1.7 μmol/L, and indirect bilirubin 6.0 μmol/L. Day 2 of admission: MRI + MRCP examination: The diameters of the common hepatic duct and common bile duct were about 6 mm. There was no normal gallbladder and cystic duct in the gallbladder fossa area. There were only oval cystic abnormal signals in the lower layer of the gallbladder fossa, about 52 mm × 30 mm in size, and the wall was slightly thick. A tortuous tubular structure running toward the gallbladder fossa was seen in the anterior superior part of the tumor, with high signal intensity on FS-T2WI and fluid signal around the cystic structure. There was a high suspicion of a free gallbladder with torsion (Figures 1E–H).

### 2.5 Therapeutic intervention

**Figure 2:**
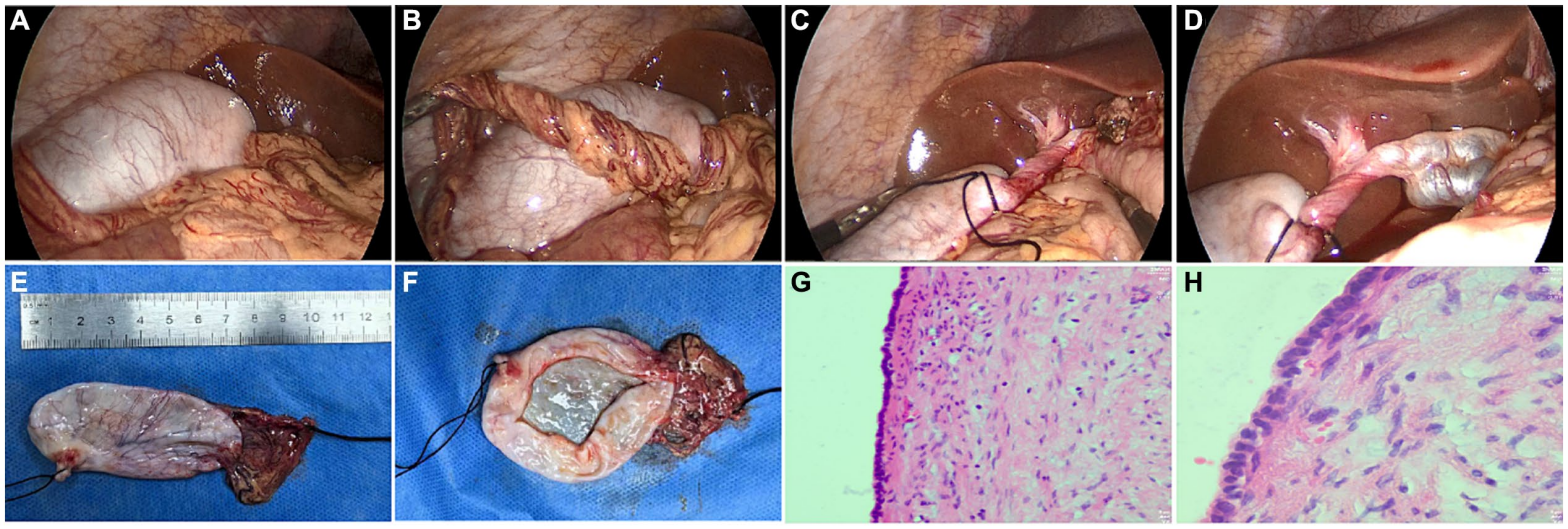
**(A)** The floating gallbladder was found during laparoscopic exploration. **(B)** Below the lower margin of the liver, numerous tortuous and infarcted omentum can be seen. **(C)** The gallbladder neck was reversed by 720 degrees counterclockwise. **(D)** Dilated cystic nodules in the uninvolved part of the neck of the gallbladder. **(E)** Gross appearance of the resected gallbladder, size about 7.0 cm × 3.0 cm. **(F)** When the gallbladder was opened, colorless transparent thin bile was found. **(G,H)** Pathological picture of the gallbladder after resection (HE staining, G × 40, H × 100).

Laparoscopic exploration was performed immediately. During the operation, the gallbladder was found to be free and slightly congested, about 7.0 cm × 3.0 cm in size, and the wall of the gallbladder was slightly thick (Figure 2A). The bottom of the gallbladder was adhered to the greater omentum, and the cystic duct was entangled with part of the greater omentum and rotated counterclockwise for 720° (Figures 2B–D). After gallbladder decompression, colorless clear thin bile was seen, and the operation was completed after reduction and torsion. The gallbladder was carefully separated and completely removed, the cystic artery was ligated, and the abdominal drainage tube was placed (Figures 2E,F).

### 2.6 Follow-up and outcomes

Postoperative abdominal pain was relieved, and routine anti-inflammatory treatment was given. Postoperative pathology showed that the wall of the gallbladder was slightly thickened with fibrosis and hyalinization, the cystic duct was slightly dilated and the discharge end was atresia. Adhesion of omental tissue was seen at the base of the gallbladder (Figures 2G,H).

## 3 Discussion

**Figure 3:**
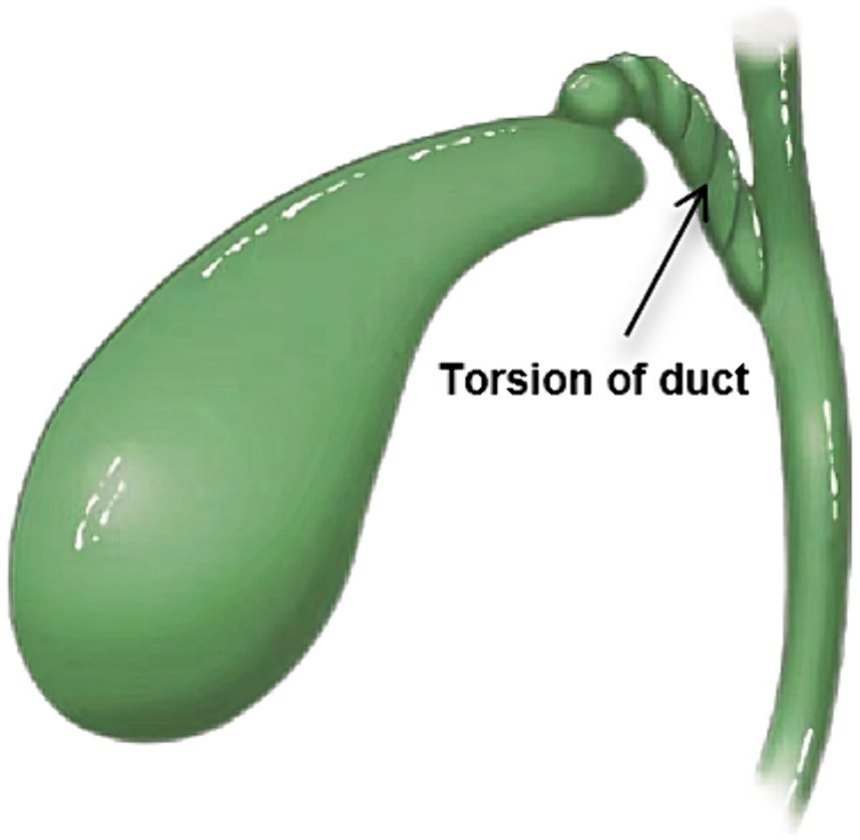
A schematic representation of the gallbladder torsion.

Acute GT (Figure 3) is more common in the elderly and adult women. The earliest adult GT can be traced back to 1898 (6, 7), which was reported by surgeon Wendel A.V. Pediatric GT is even rare, first in an 11-year-old girl reported by Daux in 1925, and only a few cases have been reported (8). So far, less than 600 cases have been reported worldwide (3), most of them are elderly and female adults. The incidence of females is higher than that of males, about 5:1 (3). It is more rare in children. There are less than 60 cases of pediatric GT reported in Chinese and foreign literature. The incidence of boys and girls is about 2.5:1 (4), and most of them are free GT. The preoperative diagnosis of acute GT is difficult because of its acute onset, atypical clinical symptoms, non-specific imaging and laboratory tests, unclear expression of children and uncooperation in physical examination, and negligence of parents to provide an exact medical history (9).

From the anatomical point of view, the gallbladder is located in the gallbladder fossa below the liver, which is connected with the liver by loose connective tissue above and covered with serosa below. It is adjacent to the right flexure of the colon and the superior flexure of the duodenum. It is relatively fixed and not prone to torsion, and only when the anatomical position of the gallbladder is variable can torsion occur (10).

Common factors for the development of GT include: (1) Congenital anatomical variation: the floating gallbladder means that the gallbladder is completely peritoneal and in a floating state, the only areas with mesentery are the gallbladder’s neck and the cystic duct, which is suspended under the liver and in a floating state (11, 12). Due to the large mobility, the gallbladder is prone to torsion along the axis of the cystic duct and the cystic artery (13), and this pediatric patient belongs to this type. (2) Acquired anatomical factors: The higher incidence of GT in the elderly is due to the gradual atrophy and reduction of the tissue structure around the gallbladder and the adipose tissue supporting the gallbladder during the aging process, the tissue degeneration and elasticity decrease and the gallbladder dropping makes the mesentery of the gallbladder longer (14, 15). In addition, peristalsis of the stomach, duodenum, and transverse colon and external factors such as physical labor and sudden changes in body position may cause GT (16).

There are two categories of GT (17). Type I: incomplete torsion, GT less than 180°, long and wide mesentery supporting the gallbladder and cystic duct; torsion can occur gradually or spontaneously lessen; Type II: complete torsion: the incomplete mesentery only supports the cystic duct when the GT exceeds 180°. This can cause the cystic duct and blood vessels to become obstructed, which can result in severe abdominal pain, infarction, gallbladder gangrene, and other potentially fatal conditions. The gallbladder can rotate either clockwise or counterclockwise during the illness, and there is not a clear correlation between the degree and direction of torsion (18). In the case of complete torsion, patients usually have a brief history of sudden and severe pain in the right upper quadrant with vomiting. In some patients, an abdominal mass can be palpable because of gallbladder swelling, with marked tenderness. There are usually no associated symptoms of toxemia or jaundice, and preoperative diagnosis is difficult. Jaundice or toxemia symptoms are typically absent, and preoperative diagnosis is difficult.

Reviewing the diagnosis and treatment process of this child, the child was admitted to our department with the symptoms of “abdominal pain and vomiting.” Physical examination revealed a mass in the right middle abdomen, accompanied by tenderness. B-ultrasound and CT results showed that the gallbladder was unclear, cystic shadow was seen in the gallbladder fossa area, common bile duct was slightly dilated, and cystic shadow was seen in the right upper abdomen, which was considered to be a benign lesion. Based on the above imaging results and clinical signs, the child was initially diagnosed with a choledochal cyst, mesenteric cyst, or cholecystitis (19). Laboratory tests showed normal white blood cells, bilirubin, and transaminase results, and the possibility of choledochal cysts and mesenteric cysts could not be ruled out. With the progression of the disease, the child’s abdominal pain worsened, and further MRCP was performed (20), which highly suspected the free gallbladder and torsion. Laparoscopic exploration was performed, and the gallbladder was counterclockwise rotated 720°, and laparoscopic LC was performed (21). The patient’s postoperative condition was stable, no complications occurred, and she was discharged safely on the 6th postoperative day.

Our department has done many cases of choledochal cyst and mesenteric cyst surgery in children, but because of the low incidence of GT in children, we have little knowledge about it. Previous studies (22) have shown that LC is a good option for the treatment of GT because there is less adhesion of the mobile mesentery to the gallbladder bed. In the last 10 years, only five pediatric cases of GT treated with LC have been reported in the English references (Table 1). Although no sufficient conclusions have been drawn, LC may be a good surgical option for GT. On the other hand, LC reveals the advantages of minimally invasive surgery: less pain, lower incidence of postoperative complications, and shorter hospital stay. Therefore, we believe that LC may be the treatment of choice for GT.

**Table 1:** Reported cases of laparoscopic cholecystectomy for gallbladder torsion in pediatric patients and with our patient added.

Author, year (ref. no.)	Age (years)	Gender	Preoperative diagnosis	Direction of rotation	Degree of torsion	Time from onset to surgery (days)	Operation time (min)	Treatment for cystic duct	Complications	Postoperative hospital stay (days)
Tandian F et al. 2015 (23)	10	M	GT	CC	360°	3	NA	NA	None	5
Uemura S et al. 2021 (24)	3	M	GT	CC	270°	3	104	Endoloop	None	6
Hoshi R et al. 2022 (8)	5	M	GT	CC	540°	NA	NA	NA	None	NA
	14	M	GT	C	360°	NA	NA	NA	None	NA
Nuyts J et al. 2024 (25)	1	F	GT	C	720°	2	65	NA	None	3
Present case	6	F	GT	CC	720°	2	95	Non-Absorbable Suture (4–0)	None	6

F, female; M, male; C, clockwise; CC, counterclockwise; NA, not available.

In our experience, the free torsion of gallbladder in children is a rare disease in clinical practice. Both clinicians and radiologists lack sufficient cognition and clinical experience of the disease. In addition, the symptoms and signs of the disease are not typical, the children are not clear and do not cooperate with the physical examination, the parents are negligent and cannot provide the exact history, and the diagnosis lacks specificity. Therefore, we should strengthen the cognition and learning of this disease. Recalling the whole process of diagnosis and treatment after operation, the application of laparoscopic exploration technology is more practical and superior in the case of unclear diagnosis.

## 4 Conclusion

In conclusion, (1) GT in children is rare. Both clinicians and radiologists should strengthen the study of this disease, improve their diagnosis and treatment level, enrich their clinical experience, and avoid missed diagnoses and misdiagnoses in the future; (2) It is recommended to give full play to the advantages of laparoscopic technology in abdominal exploration of acute abdomen, so that surgical treatment can be performed at the same time of clear diagnosis, as soon as possible to relieve the pain of patients and reduce the occurrence of complications.

## Data availability statement

The original contributions presented in the study are included in the article/supplementary material, further inquiries can be directed to the corresponding author.

## Ethics statement

The Ethics Committee of the Linyi Maternity and Child Health Care Hospital waived the need for ethics approval accordance with the local legislation and institutional requirements. Written informed consent was obtained from the minor’s legal guardian, for the publication of any potentially identifiable images or data included in this article.

## Author contributions

HR: Conceptualization, Data curation, Formal analysis, Investigation, Methodology, Project administration, Software, Validation, Visualization, Writing – original draft, Writing – review & editing. HL: Investigation, Methodology, Writing – original draft. XL: Methodology, Validation, Writing – original draft. HW: Formal analysis, Funding acquisition, Investigation, Resources, Writing – review & editing. PT: Funding acquisition, Investigation, Project administration, Resources, Software, Supervision, Validation, Visualization, Writing – original draft, Writing – review & editing.
